# The Presence of IL-17A and T Helper 17 Cells in Experimental Mouse Brain Tumors and Human Glioma

**DOI:** 10.1371/journal.pone.0015390

**Published:** 2010-10-23

**Authors:** Derek A. Wainwright, Sadhak Sengupta, Yu Han, Ilya V. Ulasov, Maciej S. Lesniak

**Affiliations:** The Brain Tumor Center, The University of Chicago, Chicago, Illinois, United States of America; UCLA and Cedars-Sinai Medical Center, United States of America

## Abstract

**Background:**

Recently, CD4^+^IL-17A^+^ T helper 17 (Th17) cells were identified and reported in several diseased states, including autoimmunity, infection and various peripheral nervous system tumors. However, the presence of Th17 in glia-derived tumors of the central nervous system has not been studied.

**Methodology/Principal Findings:**

In this report, we demonstrate that mRNA expression for the Th17 cell cytokine IL-17A, as well as Th17 cells, are present in human glioma. The mRNA expression for IL-17A in glioma was recapitulated in an immunocompetent mouse model of malignant glioma. Furthermore, the presence of Th17 cells was confirmed in both human and mouse glioma. Interestingly, some Th17 cells present in mouse glioma co-expressed the Th1 and Th2 lineage markers, IFN-γ and IL-4, respectively, but predominantly co-expressed the Treg lineage marker FoxP3.

**Conclusions:**

These data confirm the presence of Th17 cells in glia-derived CNS tumors and provide the rationale for further investigation into the role of Th17 cells in malignant glioma.

## Introduction

Glioma, which is a tumor that arises from a cell with glial lineage in the central nervous system (CNS), is a categorical designation for multiple types of glial tumors, including ependymomas, astrocytomas, oligodendrogliomas and glioblastoma multiforme (GBM). Of note, GBM is a highly malignant glioma with poor treatment options and an average lifespan of 15 months after diagnosis [1]. Recently, our lab demonstrated that a specific subset of T helper cells, CD4^+^CD25^+^ T regulatory (Treg) cells, are increased in human GBM [2], as well as various grades of astrocytoma [3]. Furthermore, Treg accumulation is recapitulated in an experimental mouse model of malignant glioma [4], [5]. Additionally, we demonstrated that Treg depletion in a mouse model of malignant glioma significantly increases lifespan [4]. Collectively, these data suggest that T helper subsets, such as Treg, are potential targets for future therapy of GBM. However, as additional T helper subsets are characterized, further investigation is required.

Recent work has identified the T helper 17 (Th17) cell subset, which is characterized as CD4^+^ T cells that produce large amounts of IL-17A (CD4^+^IL-17A^+^) [6]. Similar to Tregs, which differentiate in the presence of transforming growth factor-beta (TGF-β), Th17 differentiate in the combined presence of TGF-β and interleukin-6 (IL-6) [7]. In contrast to Tregs, Th17 require the additional cytokine, IL-23, for pathogenic activation [8]. The presence of Th17 cells has been demonstrated in autoimmune pathology [9], infectious inflammatory states [10] and tumor microenvironment [11]. Thus far, Th17 cells have not been reported to be exclusive to any one type of malignancy, as they have been observed in human ovarian-, colon- and breast-cancer, as well as melanoma [12]. However, the presence of Th17 cells in primary brain tumors has not been reported. This is particularly relevant since tumors that form in the CNS are shielded from effective T cell-mediated immunity [13], which is likely attributable due to the lack of a lymphatic system and presence of the blood-brain-barrier.

In this report, we use the GL261-cell based mouse model of malignant glioma, as well as human glioma (grade 2–4), to investigate the hypothesis that Th17 cells infiltrate brain tumors. We report that mRNA for the Th17 cytokine, IL-17A, is expressed in immunocompetent mice with glioma, but not in Rag1^−/−^ immunodeficient mice with glioma. Furthermore, mRNA for the Th17-inducing cytokines, IL-6 and TGF-β, as well as the Th17-activating cytokine, IL-23, is expressed in immunocompetent mice with glioma. Additionally, Th17 were detectable in mouse draining lymph nodes (dLN) and glioma. Importantly, mRNA expression for the Th17 cytokine, IL-17A, is increased in human glioma relative to normal human brain and Th17 cells are present in GBM. Collectively, the results suggest that Th17 cells infiltrate human and mouse glioma.

## Materials and Methods

### Animals

Six to eight-week old male C57BL/6 (stock # 000664) and Rag1KO (stock # 002216) mice were obtained from Jackson Laboratories (Bar Harbor, ME) and maintained in a specific pathogen-free facility at the University of Chicago. All surgical procedures were completed in accordance with NIH (National Institutes of Health) guidelines on the care and use of laboratory animals for research purposes. The protocol was approved by the Institutional Committee on Animal Use at the University of Chicago.

### GL261 cell line

GL261 murine glioma cells were cultured in Dulbecco's modified Eagle medium supplemented with 10% fetal calf serum, as well as streptomycin (100 mg/ml) and penicillin (100 U/ml) at 37°C in a humidified atmosphere of 95% air/5% CO2.

### Mouse intracranial injection model

Mice were anesthetized with an intraperitoneal injection of 0.1 ml of a stock solution containing ketamine HCl (25 mg/ml), xylazine (2.5 mg/ml), and 14.25% ethyl alcohol (diluted 1∶3 in 0.9% NaCl). For the stereotactic intracranial injection, the surgical site was shaved and prepared with 70% ethyl alcohol. A midline incision was made, and a 1 mm diameter right parietal burr hole, centered 2 mm posterior to the coronal suture and 2 mm lateral to the sagittal suture, was drilled. Mice were placed in a stereotactic frame and 2.5 µL PBS or 4×10^5^ GL261 cells in 2.5 µL PBS was injected intracranially with a 26-gauge needle at a depth of 3 mm. The needle was removed and the skin was sutured with 4-0 nylon thread.

### Patient samples

Resected specimens from patients who underwent operations in the Section of Neurosurgery at the University of Chicago Medical Center between 2009 and 2010 were evaluated in this study. According to the WHO classification, samples included normal brain, grade II astrocytoma, grade III oligodendroglioma, and GBM. Normal brain control tissue consisted of non-malignant tissue obtained during resection from a patient with glioma. Histological confirmation of the diagnosis for tumors was obtained in all cases by an attending neuropathologist. The tissue was collected in accordance with a protocol approved by the Institutional Review Board (IRB) at the University of Chicago.

### RNA isolation, semi-quantitative PCR and real-time PCR

Total cellular RNA from normal WT mouse brain (n = 3), 3 week post-operative (WPO) PBS-injected WT mouse brain (n = 6), 3 WPO WT mouse brain with glioma (n = 6) and 3 WPO Rag1^−/−^ mouse brain with glioma (n = 3) or normal human brain (n = 3), human astrocytoma (grade 2; n = 3), human oligodendroglioma (grade 3; n = 1) and human glioblastoma multiforme (n = 12) was isolated using the RNeasy Mini kit (Qiagen; Valencia, CA) according to the manufacturer's protocol. Equivalent amounts of RNA were reverse-transcribed with the iScript cDNA Synthesis Kit (Bio-rad Laboratories; Hercules, CA). Semi-quantitative PCR was performed using 5 uL Taq PCR Master Mix, 2.5 µM forward primer, 2.5 µM reverse primer, 2 uL RNase-free H_2_O and 1 ng input cDNA per reaction. Amplification was performed using the iCycler Thermal Cycler (Bio-Rad Laboratories) under the following conditions: 3 min at 95°C, followed by 30 cycles of 30 sec at 95°C, 30 sec at 60°C and 30 sec at 72°C, followed by 5 min at 72°C. PCR products combined with 2 uL 6X DNA Loading Dye (Fermentas Life Sciences; Glen Burnie, MD) were ran on a 2% agarose gel with the GeneRuler 100 bp DNA ladder. The gel was visualized in the ChemiDoc XRS Universal Hood II (Bio-rad Laboratories). Real-time PCR was performed using 5 uL SYBR GreenER qPCR Supermix Universal (Invitrogen; Carlsbad, CA), 0.5 µM forward primer, 0.5 µM reverse primer, 3 uL RNase-free H_2_O and 1 ng input cDNA per reaction. Amplification and detection was performed using the Opticon 2 Real-Time PCR Detector (Bio-rad Laboratories) using Opticon Monitor software (version 3.1.32; Bio-rad Laboratories) under the following conditions: 15 min hot start at 95°C, 15 sec denaturation at 95°C, 20 sec annealing of primers at 60°C, and 15 sec elongation at 72°C, for 35 cycles. Triplicate reactions were performed for all cDNA samples. For mouse and human samples, percent change in mRNA levels was calculated using the formulas ((Experimental group/PBS group) ×100)) – 100% and ((Glioma/Normal brain) ×100)) – 100%, respectively. Mouse and human primer sequences used for PCR reactions are found in Tables 1 and 2, respectively.

**Table 1:** Mouse primer sequences.

Gene symbol	GenBank accession	Forward primer 5′→3′	Reverse primer 5′→3′	Amplicon size (bp)
IL-17A	NM_010552.3	CTCAAAGCTCAGCGTGTCCAAACA	TATCAGGGTCTTCATTGCGGTGGA	130
TGF-b	NM_011577.1	CACTGATACGCCTGAGTG	GTGAGCGCTGAATCGAAA	100
IL-23	NM_031252.2	AACAGCTTAAGGATGCCCAGGTTC	ATAATGGTGTCCTTGCCCTTCACG	143
IL-6	NM_031168.1	TGGCTAAGGACCAAGACCATCCAA	AACGCACTAGGTTTGCCGAGTAGA	93
CD3e	NM_007648.4	ACCTGAAAGCTCGAGTGTGTGAGT	TGGCCTTCCTATTCTTGCTCCAGT	135
GAPDH	NM_008084.2	TCAACAGCAACTCCCACTCTTCCA	ACCCTGTTGCTGTAGCCGTATTCA	115

The gene symbols used above indicate: IL-17A  =  interleukin-17A; TGF-b  =  transforming growth factor-beta; IL-23 =  interleukin-23; IL-6 =  interleukin-6; CD3e =  CD3 antigen, epsilon polypeptide; GAPDH  =  glyceraldehyde 3-phosphate dehydrogenase.

**Table 2:** Human primer sequences.

Gene symbol	GenBank accession	Forward primer 5′→3′	Reverse primer 5′→3′	Amplicon size (bp)
IL-17A	NM_002190.2	CCACGAAATCCAGGATGCCCAAAT	ATTCCAAGGTGAGGTGGATCGGTT	144
CD3e	NM_000733.3	TTCTGGCCTGAATCAGAGACGCAT	TTCACCATGAGGCTGAGGAACGAT	150
GAPDH	NM_002046.3	TCTCCTCTGACTTCAACAGCGACA	GACAAAGTGGTCGTTGAGGGCAAT	80

The gene symbols used above indicate: IL-17A =  interleukin-17A; CD3e =  CD3 antigen, epsilon polypeptide; GAPDH  =  glyceraldehyde 3-phosphate dehydrogenase.

### Histology and immunofluorescence

At 3 WPO, WT mouse brains with glioma were flash frozen [in 62.5% n-Butyl Bromide (Fisher Scientific; Pittsburgh, PA) +37.5% 2-methylbutane (Fisher Scientific) surrounded by crushed dry ice] as previously described [14], [15], [16]. Briefly, frozen brains were sectioned at a temperature of −24°C and immersed into Tissue Teck O.C.T. Compound (Sakura Finetek USA, Inc., Torrance, CA) at 8 µm intervals and thaw-mounted onto pre-cleaned SuperFrost slides (Fisher Scientific). Sections were post-fixed with 4% paraformaldehyde, blocked for endogenous biotin for 5 min (1% H_2_O_2_ in PBS), and blocked for non-specific staining with 10% bovine serum albumin (A4503; Sigma-Aldrich; Saint Louis, MO) in PBS for 1 hr. Sections were incubated with either or the 3 antibody combinations which consisted of i) anti-GFAP-alexa fluor 488 (131-17719; Invitrogen) and biotinylated anti-CD11b (M1/70.15; Invitrogen), ii) anti-CD11c-alexa fluor 488 (N418; Ebioscience; San Diego, CA) and anti-CD11b (M1/70.15; Invitrogen) or iii) anti-CD4-alexa fluor 647 (GK1.5; Ebioscience) and biotinylated anti-FoxP3 (FJK-16S; Ebioscience) in PBS at 4°C overnight. Sections were washed extensively and incubated with streptavidin-alexa fluor 555 for 2.5 hr. Following extensive washing in PBS, sections were covered with Ultra Cruz mounting media (Santa Cruz; Santa Cruz, CA). Images of antibody-stained sections were captured using the Sp5 2-photon laser scanning confocal microscope (Leica; Mannheim, Germany). Fluorescent images were captured using the 63× objective. Additionally, 3 WPO mouse brains with glioma were stained with hematoxylin and eosin (H&E) to visualize tumor size and location. Images of H&E staining were photographed using the 1.25× objective of the Axioskop (Zeiss; Chester, VA) microscope running Openlab software.

### Surface and intracellular staining, and flow cytometric analysis

Brain and cervical (draining) lymph node cell suspensions were prepared from 3 WPO PBS-injected WT mice (n = 3), 3 WPO WT mice with glioma (n = 3) and human GBM (n = 2). Red blood cells were removed by treatment with ACK Lysis Buffer (Lonza, Walkersville, MD) for four minutes at 4°C. Cells were incubated with phorbol myristate acetate (PMA, 50 ng/ml, Sigma; St. Louis, MO) and ionomycin (500 ng/ml, Sigma, St. Louis, MO) for 4 h in the presence of GolgiBlock (1 µL/mL, Becton Dickenson (BD); Franklin Lakes, NJ). 1×10^6^ cells were stained with anti-CD4-pacific blue (RM4-5; Ebioscience) or anti-CD4-FITC (RPA-T4; BD) for mouse and human cells, respectively, for 30 min on ice. Cells were permeabilized, fixed and stained on ice using the Cytofix/Cytoperm buffer (BD) according to the manufacturer's instructions with anti-IL-17A-FITC (eBio17B7; isotype, rat IgG_2A_) biotinlyated anti-IFN-γ (XMG1.2; isotype, rat IgG1), anti-FoxP3-APC (FJK-16S; isotype, rat IgG_2A_) and anti-IL-4-PE (BVD4-1D11; isotype, rat IgG_2B_) for mouse cells or anti-IL-17A-APC (eBio64DEC17; isotype mouse IgG1) for human cells, respectively. Mouse cells were subsequently incubated with streptavidin-PerCP for 20 min on ice. The cellular frequency was determined by the LSR II flow cytometry device (Becton-Dickinson) and Flowjo analysis software (TreeStar, Cupertino, CA). Intracellular antibodies and streptavidin-PerCP were either purchased from Ebioscience of BD.

### Statistical analysis

All data are presented as means ± standard error of measurement (SEM). The results from the experiments were analyzed using GB-STAT School Pak (Dynamic Microsystems, Inc.; Silver Spring, MD). For statistical comparisons, data were analyzed using the 2-way analysis of variance (ANOVA) method, followed by *post hoc* comparisons using the Newman-Keuls test.

## Results

### An experimental mouse model of glioma

**Figure 1:**
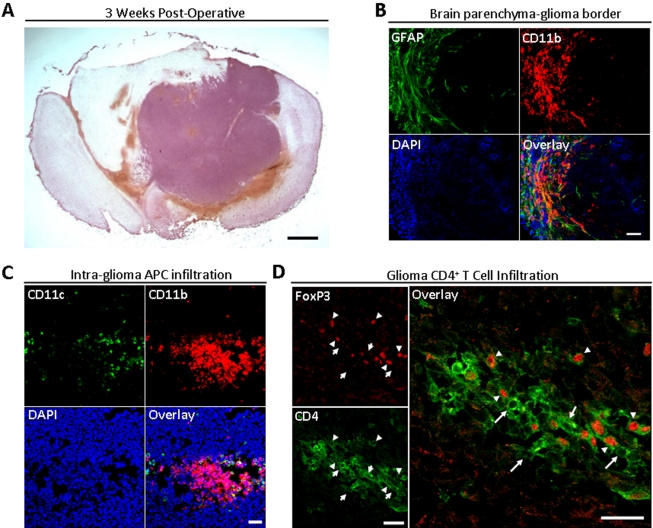
A mouse brain that received an intracranial injection of GL261 cells. At three weeks post-operatively. (A) Hematoxylin and eosin staining of mouse glioma at 3 weeks post-operatively. Scale bar  = 1 mm. (B) GFAP (green), CD11b (red) and DAPI (blue) immunofluorescence along the border between mouse brain parenchyma and glioma. Scale bar  = 50 µM. (C) CD11c (green), CD11b (red), and DAPI (blue) immunofluorescence in the mouse glioma. Scale bar  = 50 µM. (D) CD4 (green) and FoxP3 (red) immunofluorescence in the mouse glioma. Tailed arrows indicate CD4^+^FoxP3^−^ cells, while untailed arrows indicate CD4^+^FoxP3^+^ T regulatory cells. Scale bar  = 25 µM.

As shown in Fig. 1, GL261-cell based glioma takes up a large volume of intracranial space at 3 weeks post-operative (WPO) in the mouse brain. Additionally, reactive GFAP^+^ astrocytes and CD11b^+^ microglia/macrophages form a glial border around the glioma. Furthermore, CD11b^+^ and CD11c^+^ antigen presenting cells are found clustered together in the glioma. Similarly, CD4^+^FoxP3^−^ T helper and CD4^+^FoxP3^+^ T regulatory cells are found clustered together in the glioma. Thus, mouse glioma is comprised of many types of cells closely juxtaposed around- and intermixed throughout- the malignancy.

### Th17-secreted, activating and inducing cytokine levels are increased in glioma

**Figure 2:**
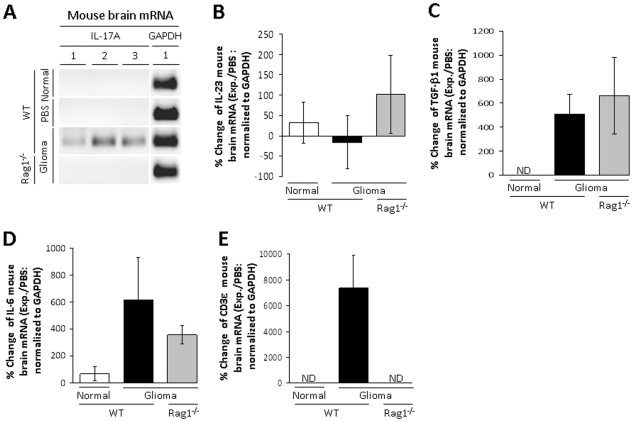
Th17-related mRNA expression levels in mouse brain with or without a GL261 cell injection. (A) PCR products for IL-17A and GAPDH from control (Normal) mouse brain or 3 week post-operative wild-type (WT) mouse brain that received an intracranial (IC) injection of PBS, WT mouse brain that received an IC injection of 4×10^5^ GL261 cells (Glioma) or recombinase activating gene 1-deficient (Rag1^−/−^) mouse brain that received an IC injection of 4×10^5^ GL261 cells (Rag1^−/−^). Individual mice are differentiated by numbers 1–3. (B) IL-23 mRNA levels are displayed as the percent change between the experimental group (Exp.) and the mouse brain that received an IC injection of PBS (PBS), normalized to GAPDH. (C) TGF-β1 mRNA levels are displayed as the percent change between the experimental group (Exp.) and the mouse brain that received an IC injection of PBS (PBS), normalized to GAPDH. (D) IL-6 mRNA levels are displayed as the percent change between the experimental group (Exp.) and the mouse brain that received an IC injection of PBS (PBS), normalized to GAPDH. (E) CD3ε mRNA levels are displayed as the percent change between the experimental group (Exp.) and the mouse brain that received an IC injection of PBS (PBS), normalized to GAPDH. Bar heights represent means (±SEM). ND = not detectable.

To determine if IL-17A mRNA is increased in mouse glioma, normal mouse brain, PBS-injected WT mouse brain, WT mouse brain with glioma and Rag1^−/−^ (lack functional T and B cells) mouse brain with glioma were analyzed (Fig. 2A). IL-17A mRNA is not detectable in normal mouse brain or mouse brain injected with PBS. However, IL-17A mRNA is detectable in glioma of WT mice, but not in glioma of Rag1^−/−^ mice. Furthermore, mRNA levels are unchanged for the Th17-activating cytokine, IL-23, in normal, WT-glioma and Rag1^−/−^-glioma mouse brain (33±51%, −16.3±65% and 102±96%, respectively). In addition, although mRNA for the Th17-inducing cytokine, TGF-β, is not detectable in normal mouse brain, it is induced in WT-glioma and Rag1^−/−^-glioma mouse brain (506±171% and 662±319%, respectively). In contrast, mRNA levels for the Th17-inducing cytokine, IL-6, is 67±53% in normal mouse brain and increases in WT-glioma and Rag1^−/−^-glioma mouse brain (618±316% and 356±67%, respectively). Finally, although mRNA for the T cell-specific complex, CD3ε, is not detectable in normal or Rag1^−/−^-glioma mouse brain, it is induced in WT-glioma mouse brain (7367±2570%). These data suggest that glioma-induced mRNA expression for the Th17 cytokine, IL-17A, as well as T cell infiltration represented by CD3ε, requires both the presence of malignancy, as well as a functional immune system. In addition, the presence of glioma appears to increase the Th17-inducing cytokines, TGF-β and IL-6.

### Th17 cells are present in glioma and draining lymph nodes

**Figure 3:**
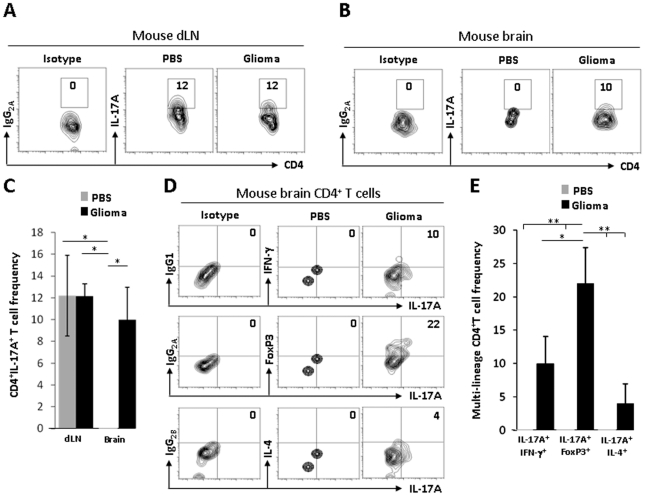
Th17 cell phenotype in dLN and mouse brain with or without a GL261 cell injection. (A) Analysis of draining lymph nodes for CD4^+^IgG2A^+^ (isotype control) or CD4^+^IL-17A^+^ cells in mice that received an intracranial (IC) injection of PBS or 4×10^5^ GL261 cells (Glioma). (B) Analysis of brains for CD4^+^IgG2A^+^ (isotype control) or CD4^+^IL-17A^+^ cells in mice that received an IC injection of PBS or 4×10^5^ GL261 cells (Glioma). (C) Average CD4^+^IL-17A^+^ cell frequency in dLNs or brains (±SEM). (D) Analysis of brains for CD4^+^IL-17A^+^IgG1^+^ (isotype control) or CD4^+^IL-17A^+^IFN-γ^+^ cells (1^st^ row), CD4^+^IL-17A^+^IgG_2A_^+^ (isotype control) or CD4^+^IL-17A^+^FoxP3^+^ cells (2^nd^ row) and CD4^+^IL-17A^+^IgG_2B_^+^ (isotype control) or CD4^+^IL-17A^+^IL-4^+^ cells (4^th^ row) in mice that received an intracranial injection of PBS or 4×10^5^ GL261 cells (Glioma). (E) Average CD4^+^IL-17A^+^IFN-γ^+^, CD4^+^IL-17A^+^FoxP3^+^, and CD4^+^IL-17A^+^IL-4^+^ cell frequency in brains (±SEM). Data are representative of at least 2 independent experiments. * and ** denotes significant differences at *p*≤0.05 and *p*≤0.01, respectively.

To determine if Th17 cells develop in glioma-draining lymph node (dLN) and are present in mouse glioma, PBS-dLN, glioma-dLN, PBS-brain and glioma-brain were analyzed at 3 weeks post-operative in WT mice. As shown in Fig. 3A-C, a two-way ANOVA for changes in CD4^+^IL-17^+^ T cell frequency revealed a main effect between mice with or without glioma [*F*(1,11) = 8.55, *p* = 0.0192]. Isotype control cells are not detectable in mouse dLN and brain. The average CD4^+^IL-17^+^ cell frequency is 12±3.7%, 12±1.1%, 0% and 10±1.5% in PBS-dLN, glioma-dLN, PBS-brain and glioma-brain, respectively. These data suggest that Th17 cells are present in the mouse dLN in response to an injury to the brain and that the level of Th17 cells is not affected by the presence of glioma. Furthermore, these data suggest that Th17 cells are present in mouse brain due to the glioma and not in response to an injury to the brain.

To determine if brain-derived Th17 cells share lineage plasticity with Th1, Treg and Th2 cells, PBS- and glioma-mouse brains were analyzed at 3 weeks post-operative in WT mice. As shown in Fig. 3D-E, a two-way ANOVA for changes in CD4^+^IL-17^+^IFN-γ^+^, CD4^+^IL-17^+^FoxP3^+^ and CD4^+^IL-17^+^IL-4^+^ T cell frequency revealed a main effect of treatment [*F*(1,17) = 24.87, *p* = 0.0003], a main effect of multi-lineage Th17 cells [*F*(1,17) = 4.33, *p* = 0.0383] and an interaction between treatment and multi-lineage Th17 cells [*F*(1,17) = 4.33, *p* = 0.0383]. Isotype control cells are not detectable in mouse brains. The average frequency of Th17 that co-expressed IFN-γ, FoxP3 and IL-4 increased from 0%, 0%, and 0% in PBS-brains to 10±4%, 22±5% and 4±3%, in glioma brains, respectively. These data suggest that glioma affects the lineage specificity of Th17 cells.

### IL-17A mRNA and Th17 cells in human glioma

**Figure 4:**
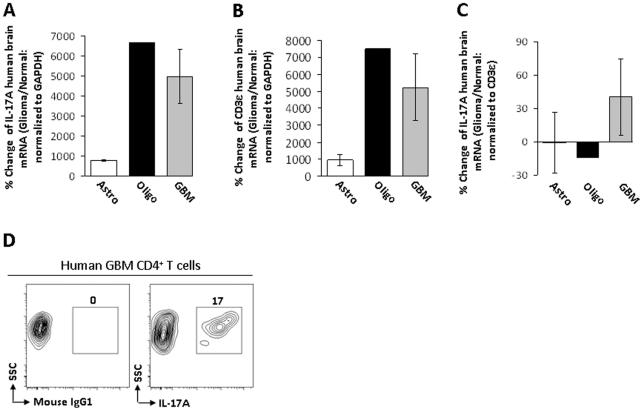
Th17-related mRNA expression levels and Th17 cells in human glioma. (A) IL-17A mRNA levels are displayed as the percent change between the glioma group (Glioma) and normal human brain (Normal), normalized to GAPDH. (B) CD3ε mRNA levels are displayed as the percent change between the glioma groups (Glioma), astrocytoma (grade 2; Astro), oligodendroglioma (grade 3; Oligo), glioblastoma multiforme (GBM) and normal human brain (Normal), normalized to GAPDH. (C) IL-17A mRNA levels are displayed as the percent change between the glioma groups (Glioma), astrocytoma (grade 2; Astro), oligodendroglioma (grade 3; Oligo), glioblastoma multiforme (GBM) and normal human brain (Normal), normalized to CD3ε. (D) Analysis of GBM for CD4^+^IgG1^+^ (isotype control) or CD4^+^IL-17A^+^ cells. Flow cytometry data are representative of at least 2 independent GBM patient specimens. Bar heights represent means (±SEM).

To determine if IL-17A mRNA is increased in human glioma, normal human brain, as well as astrocytoma (grade 2), oligodendroglioma (grade 3) and GBM were analyzed (Fig. 4A). The level of IL-17A mRNA in astrocytoma is 778±27% and increases in oligodendroglioma and GBM (6658% and 4981±1344%, respectively). Additionally, the CD3ε mRNA level in astrocytoma is 941±323% and increases in oligodendroglioma and GBM (7515% and 5231±1942%). When IL-17A mRNA levels are normalized to CD3ε mRNA levels, there is no difference between astrocytoma, oligodendroglioma and GBM (−0.7±27%, −14% and 40±35%, respectively). Finally, the frequency of CD4^+^IL-17A^+^ Th17 in GBM was determined (Fig. 4D). Isotype control cells are not detectable in GBM, while the CD4^+^IL-17^+^ cell frequency is 17%. Collectively, the data indicate that Th17 are present in human glioma and that the expression of the Th17 cytokine, IL-17A, as well as the number of T cells, is increased in oligodendroglioma and GBM, compared to low-grade astrocytoma. However, IL-17A expression appears to be dependent on T cell accumulation, since IL-17A mRNA levels were not different between types of human glioma, when normalized to CD3ε mRNA levels.

## Discussion

Collectively, the data demonstrate the presence of Th17 in mouse and human glioma. However, the presence of Th17 that co-express cytokines from other T helper lineages poses the challenge of identifying the significance and role of multi-lineage CD4^+^ T cells. Recently, it was demonstrated in mouse tumor-draining lymph nodes that the inhibition of indoleamine 2,3 dioxygenase (IDO) caused the conversion of Treg into a polyfunctional T helper phenotype with Th17 properties [17]. Additionally, Treg that expand in adenomatous polyps of colon cancer start to produce IL-17A, which results in simultaneous immunosuppression of T helper function, while promoting tumor progression through inflammation-driven mastocytosis [18]. Whether polyfunctional T helper cells have an equal level of pathogenesis in glioma has yet to be explored.

From a clinical perspective, determining the effector function of Th17 in glioma may bring novel therapeutic avenues in the field of brain tumor immunotherapy, just as determining the role of Treg in glioma did several years ago [2]–[4]. Currently, small molecule inhibitors of Th17 [19] or inhibitors of Th17 differentiation are currently being pursued [20]. In addition, Th17 inhibitors have seen mixed success in human studies, failing to control relapsing multiple sclerosis, while showing promise in patients with psoriasis and Crohn's disease [21]. Given the immunosuppressive nature of the CNS, combined with the tolerogenic [22] and multi-cellular nature of glioma, determining the role of the pro-inflammatory Th17 is important to address.

The presence of IL-17A mRNA in human glioma was recently identified through a large scale microarray analysis of Th1-, Th2- and Th17-related genes [23]. However, it was neither established whether IL-17A mRNA levels were dependent on T cell levels nor was it identified that Th17 cells were present. This study confirmed the presence of the Th17 cytokine, IL-17A, and identified the presence of Th17 cells in both human and mouse glioma. Importantly, we could not detect mRNA for the Th17-secreted cytokine, IL-17A, in Rag1^−/−^ mice with glioma, suggesting that T or B cells are required for IL-17A expression. In future investigations, we intend to identify whether IL-17A expression in glioma is solely a CD4^+^ T cell cytokine or if B cells also produce this cytokine through analysis of IL-17A mRNA levels in CD4 T cell- and B cell-deficient mice with malignant glioma. Additionally, we will identify the *in vivo* role of Th17 cells using IL-17A^−/−^ or Rag1^−/−^ mice with malignant glioma, adoptively transferred CD4^+^ T cells from IL-17A^−/−^ mice. Finally, we plan to compare the chemokine receptor repertoire of glioma-resident polyfunctional T helper cells with that of polarized T helper cell subsets. Collectively, these future experiments will determine the role of Th17 in a pre-clinical malignant glioma model, as well as determine whether these cells represent a future therapeutic target in human health.
